# Biological Effects of Add-On Eicosapentaenoic Acid Supplementation in Diabetes Mellitus and Co-Morbid Depression: A Randomized Controlled Trial

**DOI:** 10.1371/journal.pone.0049431

**Published:** 2012-11-28

**Authors:** Roel J. T. Mocking, Johanna Assies, Mariska Bot, Eugene H. J. M. Jansen, Aart H. Schene, François Pouwer

**Affiliations:** 1 Program for Mood Disorders, Department of Psychiatry, Amsterdam Medical Center, Amsterdam, The Netherlands; 2 CoRPS — Center of Research on Psychology in Somatic diseases, Department of Medical Psychology and Neuropsychology, Tilburg University, Tilburg, The Netherlands; 3 National Institute for Public Health and the Environment, Laboratory for Health Protection Research, Bilthoven, The Netherlands; German Diabetes Center, Leibniz Center for Diabetes Research at Heinrich Heine University Duesseldorf, Germany

## Abstract

**Background:**

Eicosapentaenoic acid (EPA) may reduce increased risks for (cardiovascular) morbidity and mortality in patients with diabetes mellitus (DM) and comorbid major depressive depression (MDD). Yet, effects of EPA-supplementation on biological risk factors for adverse outcomes have not been studied in DM-patients with MDD.

**Methods:**

We performed a randomized, double-blind trial (n = 25) comparing add-on ethyl-EPA-supplementation to placebo on (I) oxidative stress, (II) inflammatory, (III) hypothalamic-pituitary-adrenal (HPA)-axis, (IV) one-carbon-cycle, (V) fatty acid metabolism and (VI) lipoprotein parameters during 12-weeks' follow-up.

**Results:**

Besides increases in supplemented α-tocopherol [estimate (95% CI); 3.62 (1.14–6.11) µmol/l; p = 0.006] and plasma and erythrocyte EPA, the intervention did not influence other oxidative stress, inflammatory or one-carbon-cycle parameters compared to placebo. HPA-axis reactivity significantly decreased in the EPA-group (N = 12) [AUC_i_: −121.93 (−240.20–−3.47) min×nmol/l; p = 0.045], not in the placebo-group (N = 12). Furthermore, EPA-supplementation increased erythrocyte and plasma docosapentaenoic acid, and decreased plasma arachidonic acid (AA) concentrations [−1.61 (−3.10–−0.11) %; p = 0.036]. Finally, EPA had a multivariate influence on lipoprotein concentrations (p = 0.030), reflected by relative increases in high density lipoprotein [HDL; 0.30 (0.02–0.58) mmol/l; p = 0.039] and total cholesterol concentrations [1.01 (0.29–1.72) mmol/l; p = 0.008].

**Conclusion:**

Overall, add-on EPA-supplementation had limited effects on biological risk factors for adverse outcome in this sample of DM-patients with comorbid MDD. Besides increases in concentrations of supplemented α-tocopherol and EPA, AA decreased, and inconclusive effects on HPA-axis (re)activity and lipoprotein concentrations were observed. Therefore, further studies on the alleged beneficial effects of EPA-supplementation on biological risk factors for adverse outcome in DM-patients with comorbid MDD seem warranted, preferably using clinical outcomes such as (cardiovascular) DM-complications.

**Trial Registration:**

Controlled-Trials.com ISRCTN30877831 ISRCTN30877831

## Introduction

The prevalence of Major Depressive Disorder (MDD) in patients with type 1 or type 2 diabetes mellitus (DM) is twice that of persons without DM [1]. Importantly, in DM-patients, a diagnosis of comorbid MDD is not only associated with impaired quality of life [2], but also with an increase in (cardiovascular) complications, functional disability, and all-cause mortality [1], [3], [4]. Although the association between MDD and adverse DM-outcomes may be due to debilitating effects directly related to DM itself (e.g. comorbidities), this association between MDD and adverse DM-outcomes could also be mediated by underlying common biological mechanisms [3], [5], [6].

Both MDD and DM are independently associated with diverse endocrinological and metabolic disturbances compared with healthy controls. For example, it has been reported that MDD- and DM-patients have (I) increased oxidative stress [1], [7], together with (II) immune activation [1], [7]–[9], (III) hypothalamus-pituitary-adrenal (HPA)-axis hyperactivity [5], and (IV) one-carbon-cycle alterations [10], [11]. In addition, in both disorders, disturbances in lipid metabolism have been found with (V) lower concentrations of long chain ω3 polyunsaturated fatty acids (PUFA) [12]–[15] and (VI) altered lipoprotein concentrations [16]–[18].

These endocrinological and metabolic disturbances could be seen as a general resemblance of both biological and oxidative stress and its associated allostatic load [7], [19]. Indeed, these biological disturbances have individually been shown to act as risk factors for adverse outcomes in DM patients [11], [18], [20]–[23]. Therefore, it could be hypothesized that the biological disturbances associated with MDD add up to those in DM and thereby act as pathogenetic mediators explaining the accelerated and exaggerated development of adverse health outcomes in DM-patients with a comorbid MDD-diagnosis [24].

Normalization of these MDD-associated endocrinological and metabolic disturbances might therefore improve DM-outcomes. However, in clinical practice, treatment for MDD in DM-patients remains problematic, with low remission- and high relapse-rates [23]. Moreover, antidepressants are not always successful in the treatment of possible biological pathogenetic mediators of adverse outcomes, e.g. glycemic control, and may even have adverse effects [25], [26]. These findings emphasize the need for more effective treatment of comorbid MDD in DM-patients, in particular for the MDD-associated biological disturbances. One candidate may be PUFA from fatty fish, particularly eicosapentaenoic acid (EPA; C20:5ω3) [23], [27].

Add-on supplementation of EPA in DM-patients with MDD did not prove to be effective on MDD-symptoms [28]. However, besides merely clinical effects, EPA has been suggested to have additional effects on the above mentioned biological disturbances associated with DM and MDD. EPA is thought to lower oxidative stress levels [29]–[31], possibly through its attenuating effects on immune activation [32], [33] and HPA-axis activity [34]–[36]. This would fit with observations of beneficial influences of EPA on oxidative stress associated allostatic alterations, such as a shift of the one-carbon cycle in the remethylation direction [37] and increases in high density lipoprotein (HDL) cholesterol [32], [38]. Furthermore, EPA-supplementation alters FA-metabolism with increases in ω3- and decreases in ω6-PUFA concentrations [39]. Therefore, despite the lack of effects on MDD-symptoms, EPA might improve outcomes in DM-patients with comorbid MDD through its influence on biological risk factors for adversities associated with the comorbid MDD.

However, in contradiction to the above described lowering effects of EPA on oxidative stress, EPA-supplementation has also been suggested to increase oxidative stress. Because EPA is polyunsaturated, it is highly susceptible to peroxidation [40]. Both in and ex vivo, EPA may be subject to lipid peroxidation, conceivably resulting in potentially harmful lipid peroxidation products [40]–[42]. These lipid peroxidation products may counteract the possible beneficial effects of EPA-supplementation and thereby cause inconsistencies in the results of EPA-supplementation studies [40].

To our knowledge, the effects of EPA-supplementation on biological risk factors for adverse outcomes have not yet been studied in DM-patients with comorbid MDD. Therefore, we carried out a randomized, double-blind, placebo-controlled trial in which we performed planned secondary analyses to test the effects of add-on EPA-supplementation on (I) oxidative stress parameters, (II) immune activation, (III) HPA-axis activity, (IV) one-carbon-cycle parameters, (V) FA-metabolism and (VI) lipoprotein concentrations.

We hypothesized that EPA-supplementation would reduce (I) oxidative stress, (II) immune activation, and (III) HPA-axis reactivity. In addition, we hypothesized that EPA-supplementation would (IV) shift the one-carbon-cycle in the remethylation direction, (V) increase ω3- and decrease ω6-PUFA concentrations, and (VI) increase HDL-cholesterol.

## Methods

### Study design and participants

The study-design has been described in detail previously [28] (see supplementary material for trial protocol, translation and CONSORT checklist). In brief, we recruited patients from the VU University Medical Center diabetes outpatient clinic and through advertisements on websites, in newspapers, and patient magazines, with both MDD and DM to participate in a randomized, double-blind, placebo-controlled, balanced parallel-group study on the effects of EPA-supplementation on MDD-symptoms and biological risk factors for adverse DM-outcome. We included participants aged 18–75 years, diagnosed with DM (type 1 or 2) and MDD, and who used their current antidepressant medication for at least two months. To determine whether patients fulfilled the DSM-IV criteria for MDD we used the Composite International Diagnostic Interview (CIDI) [43]. We regarded subjects as DM-patients when they were diagnosed with DM in their medical status, used oral hypoglycemic agents and/or insulin. Exclusion criteria included pregnancy, serious comorbid disease, using fish oil supplements, consuming more than three servings of fish per week, alcohol or drug abuse, suicidal ideation, and allergy to fish, fish products or rapeseed oil. We recruited participants from April 2006 until May 2007 and performed the trial between June 2006 and July 2007. The study protocol was approved by the ethical committee of the Vrije Universiteit (VU) University Medical Center. All participants gave written informed consent.

### Intervention

Randomization occurred with computer-generated random numbers, performed by an employee of the pharmacy of the VU University Medical Center, who was not involved in the data collection and analysis. Patients were randomly allocated to 1 gram/day fish oil, containing >90% ethyl-ester EPA, or an equivalent dose of rapeseed oil and medium chain triglycerides (placebo). The EPA and placebo were packed in 500 mg soft gelatin capsules identical in appearance, and provided by Minami Nutrition, Belgium. To protect against lipid peroxidation, we stabilized the capsules with mixed tocopherols [40]. Peroxidizability differed between the EPA and placebo capsules, so different concentrations of tocopherols (Vitamin E) were added. The EPA capsules contained 11.3 g/L δ-tocopherol, 27.7 g/L ✓-tocopherol, and 3.6 g/L α-tocopherol, and the placebo capsules contained 12.8 g/L δ-tocopherol, 29.3 g/L ✓-tocopherol, and 1.7 g/L α-tocopherol. We instructed patients to consume two capsules per day for 12 weeks in addition to their anti-depressant therapy. We advised patients not to chew the capsules. Patients, the research nurse and researchers were blinded for treatment allocation until completion of the data-collection.

### Measurements

#### General

The day before starting either the intervention or placebo treatment, we conducted a baseline measurement (week 0) at the VU medical centre. Herein, we asked age, gender, educational level, cohabiting status, level of fish consumption during the last month, and use of fish oil supplementation. In addition, we obtained information on smoking habits, weight, height, DM-type, DM-treatment, and DM-complications. We collected blood samples after an overnight fast both in serum tubes and EDTA plasma tubes by venapuncture at baseline and in week 12. Both tubes were centrifuged within 2 hours after blood withdrawal. Serum samples were divided into 0.5 mL aliquots and stored at −80°C until analysis. The erythrocytes from the EDTA plasma tubes were washed two times with saline and subsequently stored at −80°C until analysis. Analyses were performed by the National Institute for Public Health and the Environment (RIVM) on the following factors. All measures were performed in serum, unless otherwise stated. Intra-assay variability was well below 8% for all measures (Table S1). The inter-assay variability was not determined because all samples have been analyzed in the same assay.

#### Oxidative stress

Since there is no single leading biomarker of oxidative stress [44], we measured a set of six parameters to get an indication of the levels of oxidative stress present in the patients. First, we measured concentrations of reactive oxygen species (ROS), as instigators of oxidative stress, using the dROMs kit from Diacron, Grosseto, Italy. Higher ROS concentrations indicate more oxidative stress. In addition, we measured concentrations of malondialdehyde, an often used biomarker of oxidative damage to lipids [7], [44], by high performance liquid chromatography using a kit from Chromsystems, Munich, Germany. For malondialdehyde, higher concentrations also indicate more oxidative stress damage. Furthermore, we included several markers of anti-oxidative capacity. First, we analyzed the gluthathione system in erythrocytes, the most important anti-oxidative defense system [44], and expressed it in the ratio of oxidized glutathione to reduced glutathione (GSSG/GSH). A higher ratio reflects more oxidative stress. Total glutathione (GSHtot) and oxidized gluthation (GSSG) have been measured after deproteinization by using glutathione reductase and DTNB. GSSG is determined after derivatization of GSH by 2-vinylpyridine on an autoanalyser. In addition, we measured vitamin E concentrations by reversed phase HPLC with fluorescence detection, in the form of ✓-tocopherol (the major form in human diet) and α-tocopherol (the most extensively studied form) [45]. Vitamin E is the major membrane antioxidant necessary for the renewal of cells and to inhibit inflammatory lesions [7], [44], [46]. Finally, we measured activity of superoxide dismutases (SOD) in erythrocytes, using a kit from Randox Antrim, GB. SOD detoxificate superoxide, and thereby contribute to the antioxidant defense [7]. Higher SOD-activity is consistent with high levels of oxidative stress.

#### Inflammation

As indication of inflammatory activity in our patients, we assessed three inflammatory biomarkers. We measured concentrations of C-reactive protein (CRP) by a dedicated high-sensitive kit from Beckman-Coulter, Woerden, the Netherlands. We determined interleukin-6 (IL-6) and tumor necrosis factor-α (TNF-α) using high-sensitive enzyme immune-assay kit from R&D Systems, Abingdom, GB. These clinically relevant markers of inflammation are associated with complications in DM [21] and related to MDD [9], [47].

#### One-carbon-cycle

To assess one-carbon-cycle metabolism [10], we measured concentrations of vitamin B_12_, folate and homocystein, using a kit from Dialab, Vienna, Austria.

#### Fatty acids

In erythrocyte phospholipids, we measured EPA, docosapentaenoic acid (DPA; 22:5ω3) and docosahexaenoic acid (DHA; 22:6ω3) by gas chromatography (GC-3900, Varian Assoc., Palo Alto, USA). In addition, we measured concentrations of 33 FA in the phospholipid fraction in serum, including EPA, DHA, arachidonic acid (AA; C20:4ω6), and nervonic acid (NA; C24:1ω9). The FA-content was expressed as percentage of the total fatty acids present in the chromatogram. Subsequently, we calculated three indices to delineate important structural FA-characteristics [48]. These indices were the unsaturation index (UI), chain length index (CLI) and peroxidation index (PI), according to previously defined formulas [49]. The UI provides information about the mean number of double bounds per FA, the CLI denotes the mean FA-chain length, while the PI indicates the mean FA susceptibility to oxidative stress.

#### Lipoproteins

We measured cholesterol concentrations in low and high density lipoproteins (LDL, HDL) together with total cholesterol, using dedicated kits from Beckman-Coulter, Woerden, the Netherlands. In addition, we calculated the total/HDL cholesterol ratio.

#### HPA-axis

We measured HPA-axis activity by determining salivary cortisol by enzyme immunoassay using a kit from DSLabs, Beckman-Coulter, Germany. Subjects collected saliva using salivettes at awakening, 30 minutes, 45 minutes and 1 hour thereafter, also at baseline and week 12. After centrifuging, the clear saliva samples were stored at −20°C until analysis. For practical reasons, the cortisol concentrations at awakening, 30 and 45 minutes, and 1 hour thereafter for both baseline and follow-up measurements were reformulated in area-under-the-curve_ground_ (AUC_g_; total hormonal output) and area-under-the-curve_increase_ (AUC_i_; changes over time) scores in accordance to previously described formulas [50].

### Statistical analyses

We calculated sample size for the study using G*Power 3.0.10, for the within-between interaction for the original study's primary outcome variable – MDD-symptom severity – as described previously [28]. As seven repeated measures of MDD-symptom severity were obtained, 10 patients had to be randomized to each intervention group to detect a standardized effect size of f = 0.25 for the primary outcome variable (power 80%, two-sided *α* = 0.05, correlation between repeated measures = 0.6 and non-sphericity correction ε = 0.6). We assumed a drop-out of 20%, so 25 patients were included. In the present investigation our biological outcome variables were measured twice. Hence, the reported sample size calculation does not apply to the present investigation. Therefore, we recalculated the power for the biological outcome variables in analogy to the power calculations for the primary outcome variable, by changing the number of measurements in G*Power from 7 to 2. With 80% power and the assumed 20% drop-out, this resulted in a detectable effect size of 0.30 instead of 0.25 for the biological outcome variables under investigation in the present study.

Intention to treat analyses were conducted. Outcome variables that were not normally distributed were log-transformed to achieve normality. Because our pathophysiological characteristics (i.e. oxidative stress, inflammation, one-carbon-cycle, fatty acids and lipoproteins, HPA-axis) consisted of multiple biomarkers, we used repeated measures multivariate analyses of variance (MANOVA) to test the effects of EPA-supplementation on these outcomes. If the multivariate treatment×time interaction test statistic was significant, we performed follow-up linear mixed models for the individual variables. In case a genuine effect exists, these subsequent mixed model analyses for the individual variables are thought to be protected by the initial MANOVA [51], [52]. From the mixed model analyses, we derived effect estimates for the treatment×time interaction effects, together with 95% confidence intervals in order to distinguish between absence of effect and absence of evidence of effect. The large number of biomarkers tested makes it difficult to exclude type I errors, therefore, results should be interpreted with caution. However, because our study is of an explorative nature, we did not additionally correct for multiple testing [52]. Furthermore, we reported indices of effect sizes (i.e. partial η^2^ values and Cohen's d) where appropriate. Partial η^2^ values represent the proportion of the variance in the dependent variable that can be explained by the independent variables and were derived from repeated measures MANOVA; 0.01 indicates a small, 0.06 a moderate, while 0.14 indicates a large effect. However, partial η^2^ has a tendency to overestimate effect size, and thus should be interpreted with caution. Cohen's d was used to describe to magnitude of differences derived from paired samples t-tests, when comparing the before and after treatment states. For Cohen's d, 0.2 indicates a small, 0.5 medium and 0.8 a large effect. All analyses were performed in PASW statistics 18.0 (SPSS, Inc., 2009, Chicago, IL).

## Results

**Figure 1:**
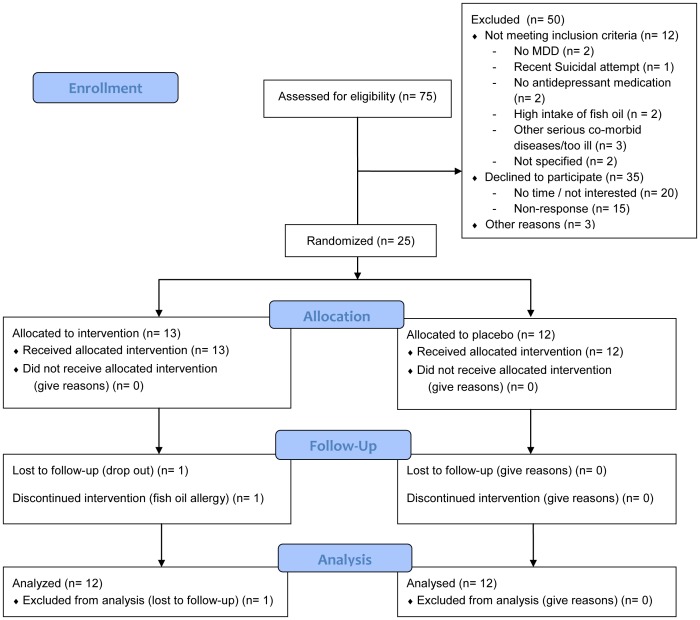
Flow diagram.

The participant flow, compliance and adverse events in this trial have been described previously [28]. In brief, 75 persons initially volunteered to participate in the study. Twenty persons were not interested or had no time, 15 did not respond, and 12 were excluded because they did not meet inclusion criteria (see Figure 1 for CONSORT flowchart). For three persons, reasons of non-participation were unknown. Twenty-five patients were randomized to either EPA (n = 13) or placebo (n = 12), consisting of 13 women and 12 men with a mean age of 54 (±11) years. At baseline, the two intervention groups did not differ significantly with regard to age, gender, smoking status, body mass index, fish consumption, DM-type, DM-complications, DM-treatment, HbA1c, MDD-severity, and oxidative stress, inflammatory, HPA-axis, one-carbon cycle, and lipoprotein parameters, indicating successful randomization (Table S2). For the 33 baseline FA-concentrations and 3 indices, the two intervention groups also did not differ, except for erythrocyte membrane DPA (table 1, p = 0.026) and plasma phospholipid 16:3ω4 (EPA-group 0.12±0.02; placebo-group 0.15±0.04; p = 0.034). One EPA-patient showed an allergic reaction and discontinued using EPA, but no other severe adverse events were reported. Both baseline and 12 weeks follow-up saliva and blood samples were available for 19 and 24 patients, respectively. Baseline and follow-up means of the concentrations of the biological parameters for the intervention and placebo group are given in table 1.

**Table 1:** Means and standard deviations of several biological parameters in patients with diabetes mellitus and co-morbid depression (n = 24) before (baseline) and after (follow-up) 12-week randomized add-on supplementation of either ethyl-eicosapentaenoic acid or placebo.

Parameter	Baseline EPA	Follow-up EPA	Baseline placebo	Follow-up placebo
ROS, U/ml	440.25±84.33	424.33±90.45	395.67±51.49	392.25±58.62
MDA, µmol/l	0.52±0.21	0.49±0.19	0.47±0.15	0.42±0.18
GSSG/GSH ratio	0.34±0.12	0.31±0.10	0.35±0.13	0.26±0.07^b^
✓-tocopherol, µmol/l	3.68±1.55	5.26±1.63	4.52±1.27	6.51±3.60^b^
α-tocopherol, µmol/l	26.11±3.51	27.24±3.33^c^	28.91±3.65	26.42±3.37^b^
SOD, U/µmol Hb	11.51±6.94	12.46±6.34	13.10±4.16	10.70±1.14
CRP, mg/l^a^	3.26 (6.08)	3.92 (6.90)	3.79 (4.90)	3.47 (6.50)
IL-6, pg/ml^a^	2.20 (2.91)	2.18 (1.09)	2.20 (1.84)	2.24 (1.60)
TNF-α, pg/ml^a^	2.11 (0.66)	1.97 (0.92)	2.32 (1.56)	2.09 (1.38)
AUC_g_	537.58±227.54	424.42±115.39	488.18±255.70	526.43±315.18
AUC_i_	145.58±167.45	23.75±166.20^d^	148.58±229.44	136.42±248.35
Vitamine B_12_, pg/ml^a^	268.50 (141.25)	299.50 (99.75)	276.00 (118.00)	296.00 (173.50)
Folate, nmol/l^a^	13.20 (7.71)	12.53 (9.07)	13.29 (8.84)	16.66 (12.56)
Homocystein, µmol/l	13.31±4.80	13.75±4.95	16.16±5.34	16.17±5.12
Erythrocyte EPA, %	0.54±0.17	1.69±0.56^c^^,^ e	0.66±0.20	0.61±0.19
Erythrocyte DPA, %	2.17±0.39^f^	3.50±0.71^c^^,^ e	2.57±0.46	2.55±0.44
Erythrocyte DHA, %	4.47±0.83	4.35±0.93	4.75±1.19	4.79±1.01
Peroxidation index	1.12±0.14	1.16±0.13	1.11±0.06	1.12±0.11
Chain length index	18.07±0.09	18.08±0.10	18.06±0.07	18.07±0.07
Unsaturation index	1.39±0.09	1.40±0.07	1.37±0.04	1.38±0.07
Linoleic acid, %	21.26±4.10	20.89±3.15	21.04±1.97	20.99±2.78
Arachidonic acid, %	10.82±2.23	9.91±2.02^g^	9.86±1.78	10.45±2.47
α-linolenic acid, %	0.14±0.07	0.18±0.08	0.19±0.09	0.18±0.08
EPA, %	0.88±0.42	2.24±1.06^d^^,^ e	0.96±0.23	0.87±0.34
DPA, %	0.90±0.27	1.34±0.46^c^^,^ g	1.05±0.25	1.02±0.28
DHA, %	3.07±1.03	2.85±0.85	3.17±0.96	3.21±0.87
Oleic acid, %	8.19±0.77	8.05±0.60	8.85±2.02	8.78±1.71
Nervonic acid, %	1.17±0.42	1.22±0.30	1.24±0.23	1.38±0.36
LDL cholesterol, mmol/l	2.65±0.85	2.69±1.07	3.20±1.10	2.69±0.74
HDL cholesterol, mmol/l	1.17±0.30	1.27±0.64	1.42±0.53	1.23±0.38^b^
Total cholesterol, mmol/l	4.60±0.84	4.76±1.17	5.38±1.20	4.53±0.89^b^
Total/HDL cholesterol ratio	4.16±1.29	4.20±1.65	4.10±1.16	3.83±0.78

a Median (Inter-quartile range), significance non-parametrically tested.

b Significantly different from baseline concentrations in the placebo-group (*P*<.05).

c Significantly different from baseline concentrations in the EPA-group (*P*<.001).

d Significantly different from baseline concentrations in the EPA-group (*P*<.05).

e Significantly different from follow-up concentration in the placebo-group (*P*<.001).

f Significantly different from baseline concentrations in the placebo-group (*P*<.05).

g Significantly different from follow-up concentration in the placebo-group (*P*<.05).

### Oxidative stress

The multivariate effect of the treatment×time interaction on the six selected oxidative stress parameters was significant (*F* = 2.898; p = 0.039; partial η^2^ = 0.506), indicating an effect of the supplementation on the overall course of the parameters. Subsequent linear mixed model analyses indicated that this multivariate result was due to an increase in α-tocopherol in the EPA-supplementation group relative to the placebo group (table 2). The courses of the other 5 measures of oxidative stress were not significantly altered by EPA-supplementation in linear mixed model analyses (table 2).

**Table 2:** Linear mixed model treatment×time interaction effects for selected biological parameters from significant repeated measures MANOVA's in diabetes patients with MDD during 12-week randomized add-on supplementation of either ethyl-eicosapentaenoic acid or placebo (n = 24).

Parameter	Estimate	SE	95% CI	t	P
ROS, U/ml	−12.50	17.55	−48.91–23.91	−0.71	.484
MDA, µmol/l	0.018	0.089	−0.166–0.201	0.20	.845
GSSG/GSH ratio	0.077	0.048	−0.022–0.18	1.61	.121
✓-tocopherol, µmol/l	−0.41	0.90	−2.28–1.47	−0.45	.658
α-tocopherol, µmol/l	3.62	1.20	1.14–6.11	3.02	.006
SOD, U/µmol Hb	3.19	3.05	−3.11–9.49	1.05	.306
Erythrocyte EPA, %	1.19	0.13	0.91–1.47	8.85	1.01×10^−8^
Erythrocyte DPA, %	1.34	0.13	1.08–1.61	10.5	4.26×10^−10^
Erythrocyte DHA, %	−0.16	0.15	−0.47–0.15	−1.05	.304
Linoleic acid, %	−0.28	0.83	−2.00–1.44	−0.34	.737
Arachidonic acid, %	−1.61	0.72	−3.10–−0.11	−2.24	.036
α-linolenic acid, %	0.05	0.03	−0.02–0.12	1.61	.126
EPA, %	1.54	0.31	0.89–2.19	4.92	7.00×10^−5^
DPA, %	0.46	0.08	0.29–0.63	5.53	1.39×10^−5^
DHA, %	−0.25	0.33	−0.94–0.43	−0.78	.445
Oleic acid, %	−0.04	0.48	−1.05–0.96	−0.09	.933
Nervonic acid, %	−0.09	0.11	−0.32–0.14	−0.79	.440
LDL cholesterol, mmol/l	0.56	0.34	−0.15–1.27	1.63	.117
HDL cholesterol, mmol/l	0.30	0.14	0.02–0.58	2.19	.039
Total cholesterol, mmol/l	1.01	0.34	0.29–1.72	2.92	.008
Total/HDL cholesterol ratio	0.31	0.27	−0.24–0.87	1.18	.251

### Inflammation

There was no significant multivariate effect of the treatment×time interaction on the 3 inflammatory markers (*F* = 0.156; p = 0.924; partial η^2^ = 0.025). Therefore, linear mixed models were not indicated. This shows that EPA-supplementation had no effect on inflammatory markers in our sample of DM-patients with MDD, compared to placebo (for reasons of completeness, univariate mixed model results of non-significant multivariate biomarkers are shown in Table S3).

### HPA-axis

**Figure 2:**
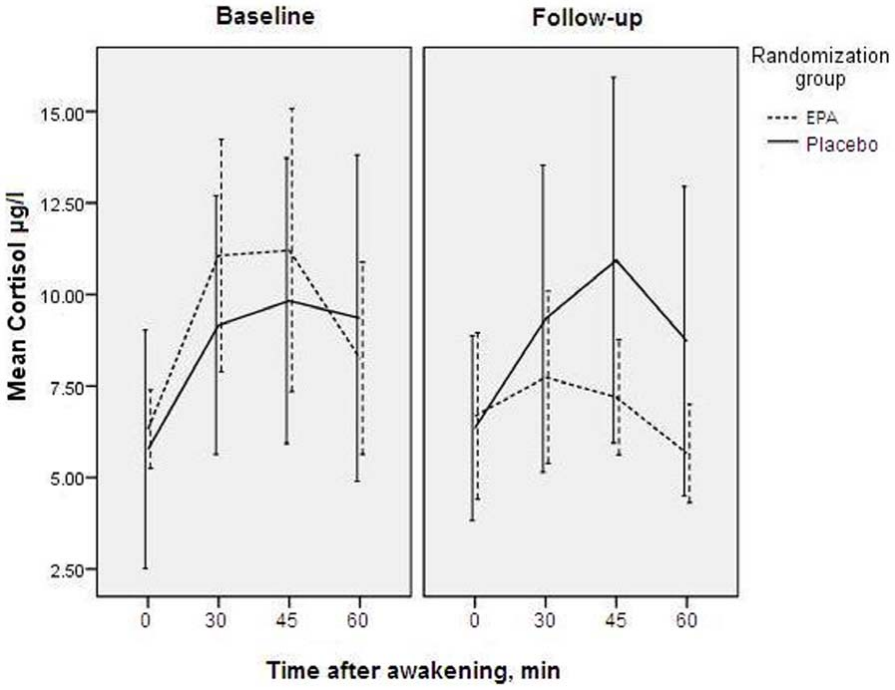
Effects of EPA on cortisol. Cortisol concentrations at baseline and 12-week follow-up, in diabetic patients with comorbid depression (n = 19) randomized to add-on supplementation of either ethyl-eicosapentaenoic acid or placebo. Error bars indicate 95% confidence intervals.

The multivariate treatment×time interaction for the AUC_g_ and AUC_i_ was not significant (*F* = 1.310; p = 0.297; partial η^2^ = 0.141; Figure 2). However, the high correlation between the two outcome variables (Pearson's *r* = .607; p = 0.006), and large variance naturally present in cortisol concentrations may have increased the chance of type II errors in MANOVA. Therefore, we performed secondary analyses, in which we additionally tested the difference between follow-up and baseline concentrations in each intervention arm using paired samples t-tests. The follow-up AUC_g_ was not significantly different from the baseline measures, in both the EPA-supplementation-group (*t* = −1.505; df = 8; p = 0.171; Cohen's *d* = −0.709) and the placebo-group (*t* = 0.486; df = 9; p = 0.639 Cohen's *d* = 0.217). However, the AUC_i_ showed a significant decrease in the EPA-group over time (*t* = −2.374; df = 8; p = 0.045; Cohen's *d* = −1.119), compared with a non-significant change in the placebo-group (*t* = −0.247; df = 9; p = 0.810; Cohen's *d* = −0.110).

### One-carbon-cycle

The multivariate test for the effect of the treatment×time interaction was non-significant for the three one-carbon-cycle factors: vitamin B_12_, folate and homocysteine (*F* = 1.212; p = 0.331; partial η^2^ = 0.154). Therefore, linear mixed models were not indicated.

### Fatty acids

The multivariate treatment×time interaction-effect on the three indices (UI, CLI and PI) reflecting main characteristics of FA-metabolism was not significant (*F* = 1.355; p = 0.288; partial η^2^ = 0.184). However, the multivariate treatment×time interaction-effect for the FAs in the phospholipid fraction [oleic acid (OA; C18:1ω9), α-linolenic acid (ALA; C18:2ω3), linoleic acid (LA; C18:2ω6), arachidonic acid (AA; C20:4ω6), EPA, DPA, DHA, and nervonic acid (NA; C24:1ω9)] was significant (*F* = 5.151; p = 0.005; partial η^2^ = 0.760). Subsequent linear mixed models showed that this effect consisted of a significant treatment×time interaction for AA, EPA, and DPA (table 2). This indicates that EPA-supplementation particularly increased both EPA- and DPA-concentrations, while it also decreased AA-concentrations compared with the placebo-group. In the erythrocyte, a similar pattern was observed with a significant multivariate treatment×time interaction for EPA, DPA and DHA (*F* = 35.249; p = 3.545×10^−8^; partial η^2^ = 0.841). Subsequent linear mixed models also showed increases in EPA and DPA (table 2). Interestingly, DHA-concentrations did not significantly change over time in the EPA-group compared with the placebo-group in both plasma and erythrocyte phospholipids (table 2).

### Lipoproteins

The multivariate treatment×time interaction-effect on total cholesterol, LDL and HDL was significant (*F* = 3.648; p = 0.030; partial η^2^ = 0.354), indicating a significant effect of EPA on changes in lipoprotein concentrations over time compared with placebo. Subsequent linear mixed models revealed that this effect consisted of a relative increase in HDL and total cholesterol concentration in the EPA-group (table 2). Differences in LDL-concentrations over time did not significantly differ between the two groups (table 2). The multivariate treatment×time interaction-effect was not significant for the total/HDL cholesterol ratio.

## Discussion

In this randomized, double-blind, placebo-controlled trial, we found limited effects of add-on EPA-supplementation on measured biological risk factors in a sample of DM-patients with comorbid MDD. First, supplementation relatively increased α-tocopherol concentrations, but EPA had no effects on other parameters of oxidative stress compared to placebo. Second, changes in inflammatory markers were not significantly different between the two groups. Third, reactivity of the HPA-axis diminished in the EPA-group, while, fourth, EPA had no significant influence on one-carbon-cycle parameters. Fifth, though FA-indices were not significantly altered by EPA-supplementation, individual FA-concentrations were influenced with increases in EPA- and DPA-concentrations and decreases in AA. Finally, sixth, EPA increased HDL and total cholesterol relative to placebo. To the best of our knowledge, this is the first study investigating the biological effects of EPA in DM-patients with comorbid MDD.

### Oxidative stress

Our results suggest that supplementation significantly increased α-tocopherol, consistent with previously reported increases in α-tocopherol after supplementation in MDD [53]. This increase likely reflects the higher α-tocopherol content in the EPA-capsules compared to placebo, necessary to prevent EPA from lipid peroxidation [40]. EPA had no effects on ROS, malondialdehyde, the GSSG/GSH ratio and SOD activity, neither consistent with increasing nor decreasing effects of EPA-supplementation on oxidative stress in this sample. Data regarding the effects of EPA on oxidative stress in other populations available thus far remain inconclusive [54], with some studies reporting lowering effects [29], and others absence of [37], or even increasing effects [40], [41]. Nevertheless, particularly our finding of no increasing effects of EPA supplementation on malondialdehyde, indicates that the supplemental EPA is not subject to lipid peroxidation. The possibility that harmful lipid peroxidation products are formed is thereby reduced, so our results form no contraindication for future supplementation studies in depressed DM-patients that can further clarify these issues.

### Inflammation

Although EPA is thought to suppress inflammatory responses [55], we did not find any differences in changes of CRP, IL-6, and TNF-α compared to placebo. For CRP, a suppressive effect of EPA was found in DM-subjects [32], but not consistently [29]. We are not aware of any studies on the effects of EPA on CRP in MDD. For IL-6, our findings are in agreement with studies on subjects with either DM [29], [32] or MDD [36]. With regard to TNF-α, our findings of a lack of effect of EPA are similar to some [29], but not all previous research in DM [32], while MDD-patients have not been studied. These inconsistent findings could be because some studies are not placebo-controlled [32], which may have affected results. The absence of effects of EPA on inflammatory markers in our and previous placebo-controlled trials could be explained by anti-depressant and/or diabetic medication patients received, which could have normalized inflammatory marker concentrations [56]. This is supported by relatively modest baseline concentrations of inflammatory markers in our sample. In that case, the possibility for EPA to normalize concentrations would be reduced.

### HPA-axis

Analyses of variance showed no differences in the courses of the HPA-axis measures between placebo and control groups. This could indicate that EPA had no effect on the HPA-axis in this sample. However, as noted previously, this absence of effects might also be due to type II errors [57], because of strong correlations between outcome variables and large natural variability in cortisol concentrations. Our findings that in the EPA group, AUC_i_ significantly decreased (with a large effect size), compared to no significant changes in the placebo group (Figure 2), might also hint in this direction [57]. A decreasing effect of EPA on the AUC_i_ would be in accordance with previously reported effects of EPA-supplementation on cortisol in MDD [36]. DM-patients have not been studied, but in healthy subjects, fish oil (rich in EPA) supplementation caused blunted cortisol concentrations after mental stress [34] or an endotoxin challenge [58], in accordance with our results and those in rats [59]. However, overall, effects of EPA-supplementation on cortisol in this sample of DM-patients with comorbid MDD remain inconclusive.

### One-carbon-cycle

In our sample of DM-patients with MDD there was no evidence for an effect of EPA-supplementation on one-carbon-cycle parameters. Data on MDD-patients are not available. In DM-patients, previous findings of homocysteine decreasing effects of ω3 PUFA supplementation contradict our findings [37], [60], as do findings in healthy women [31]. Apart from differences in study population, this could be due to the use of other supplementation-products (ethyl-EPA vs. ω3 PUFA in general). In addition, increases in homocysteine concentrations after fish oil supplementation were observed in healthy subjects, without effects on folate or vitamin B_12_; these latter two findings in accordance with our study [61].

### FA-concentrations

While EPA did not affect overall structural FA-indices (UI, CLI, PI), it increased EPA-concentrations, as expected and reported in previous papers [28], [62], and also DPA-concentrations. This corresponds with previous findings in DM or MDD [39], [53], [63], [64], and also suggests good study compliance. Supplementation also decreased AA-concentrations compared with the placebo-group, this is in agreement with some [39], but not all previous findings in DM [63], while in MDD it was shown that supplementation decreased total ω6 FAs [53]. The inconsistencies between our findings and those of Rondanelli et al. considering the effect of EPA on AA [63], may be due to differences in study population (elderly subjects with MDD or dysthymia vs. subjects with MDD and DM), or intervention characteristics (1.67 gram EPA and 0.83 gram DHA vs. 1 gram >90% ethyl-ester EPA). Our observation of a lack of effect of EPA on DHA corresponds to a previous report in DM [64], but not in MDD [53]. This lack of effect, contrary to our hypothesis, could possibly imply that DHA-concentrations are, possibly adaptatively, endogenously regulated in these patients [14], and therefore less affected by increased dietary availability of the DHA precursor EPA [65]. Considering the association between DHA and brain derived neurotrophic factor (BDNF) [66], this is consistent with the absence of effects of EPA on BDNF in our sample [62]. The observed increases in DPA could resemble the difference in baseline concentrations between the EPA and placebo group. However, because we compared baseline values of 33 FA and 3 indices, and groups were based on randomization, this could be due to chance. In addition, both erythrocyte and plasma DPA concentrations showed a marked increase in the EPA-group. Therefore, these increases in DPA-concentrations are likely caused by the EPA-supplementation, and could possibly function as a storage form for later conversion to DHA [67].

### Lipoproteins

EPA increased HDL and total cholesterol, without significant differences in LDL. These findings are not consistent with a meta-analysis in DM-patients, which reported non-significant HDL increases, absence of changes in total cholesterol and increases in LDL [68]. However, a randomized controlled trial in MDD patients also showed HDL increases after fish oil supplementation [53]. So, it could be hypothesized that EPA has a different effect on lipoprotein concentrations in MDD than in DM. The increase in HDL concentrations in the EPA-group relative to placebo coincides with increases in total cholesterol concentration, also reflected by absence of a treatment effect on the total/HDL cholesterol ratio. So, our data do not allow definitive conclusions; further research on the effects of EPA-supplementation on concentrations of oxidized forms of lipoproteins may yield additional relevant results.

### Limitations and strengths

Our study has several limitations. First, power was initially calculated to detect effects of EPA on MDD-symptom severity for which 7 repeated measurements were available [28], instead of the 2 repeated measurements (baseline and follow-up) for the biological outcomes in the present study. In the initial power calculations, the power was 80% to detect an effect size of 0.25 [28]. Considering the 2 repeated measures for the biological outcomes in the present study, calculated power was 80% for an effect size of 0.30. The decreased power due to less repeated measures could have resulted in non-detection of true effects with a small effect size. Nevertheless, the remaining power was adequate to detect effects with a medium to large effect size. In addition, the relatively small number of drop-outs (less than the expected 20%) resulted in enough power to detect effects with effect sizes between 0.25 and 0.30 for most outcome variables.

Second, the DM-group included both type 1 and type 2 DM-patients. It may be that the effects of EPA on the measured biological factors in our patients with DM and MDD are modulated by DM-subtype. Our results are therefore only generalizable to the whole population of patients with DM and MDD, not specifically to only those patients with DM type 1 or type 2.

Third, the absence of effects of EPA on markers of inflammation and the one-carbon-cycle may be because the dose was not sufficient [69], the follow-up time was not long enough, or EPA exerts its effects on still other markers than those we selected [55].

Fourth, the intervention capsules in our study contained no DHA. Although EPA is thought to be more effective for treatment of depressive symptoms [23], [27], it could be that the effects of fish oil on measured biomarkers are mainly caused by DHA. Future studies using different concentrations of EPA in combination with DHA could further clarify this issue.

Our study also has its strengths. To our knowledge, this is the first study on the biological effects of EPA-supplementation in DM-patients with comorbid MDD. This is clinically relevant because these diseases have a large contribution to the global burden of disease [4]. In addition, there was only a small loss to follow-up. Also, because EPA was not effective in diminishing MDD-symptoms in this population [28], effects of EPA have to be directly related to its metabolic actions, not modulated by effects on the comorbid MDD. Furthermore, we were able to investigate several aspects of the biological influence of EPA at the same time. Finally, the add-on randomized, double-blind, placebo-controlled trial-design provided the opportunity to assess the preventive causal effects of EPA on clinically relevant risk factors for adverse outcome in a naturalistic setting.

### General considerations

Besides increases in the supplemented products (α-tocopherol and EPA), the intervention increased DPA, and decreased AA concentrations compared to placebo. Additionally, EPA had inconclusive effects on HPA-axis (re)activity and lipoprotein concentrations. There were no changes in oxidative stress and inflammatory markers or one-carbon metabolism. In addition, significance levels of effects would not have survived strict correction for multiple testing. Overall, this points to a limited influence of EPA on risk factors for adverse outcome in this sample of patients with DM and comorbid MDD.

Increases in the supplemented products (α-tocopherol and EPA) and decreases in AA are generally considered beneficial [23], [70], but it remains to be determined whether exogenously induced changes have similar effects. It could be hypothesized that the observed decreases in AA and increases in EPA steered the eicosanoid precursor supply in an anti-inflammatory direction [24], [55]. This would diminish oxidative stress, and the associated allostatic response. Alternatively, it could be hypothesized that the improvement in allostatic load associated disturbances is mediated through beneficial effects of EPA on transcription factors, e.g. peroxisome proliferator-activated receptor (PPAR) and sterol-responsive-element binding protein (SREBP) [71]. Through these effects EPA could improve metabolic pathways, and for example reduce insulin resistance and its associated oxidative stress [72]. However, since we found no effects of EPA on inflammatory, other oxidative stress, or one-carbon metabolism biomarkers, it seems that changes in concentrations of supplemented products did not result in alterations in these allostase-associated potential pathophysiological pathways [19]. On the other hand, EPA itself is susceptible to oxidative stress, and harmful (non-)enzymatic lipid peroxidation products may be formed, counteracting the possible beneficial effects [40]–[42], [73], [74]. Our finding of no increases in the lipid peroxidation product MDA, however, limits this risk.

EPA supplementation had no effect on mood in this sample [28], and had limited effects on biomarkers for adverse outcome. It may be possible that modulation of these risk factors would actually be effective on both mood and adverse DM outcome, but EPA supplementation may not be the optimal treatment to affect these biomarkers. This possibility would still be in accordance with hypothetical models of common pathophysiological pathways underlying the relationship between DM and MDD (e.g. inflammation, HPA-axis hyperactivity) [1], [3], [23], but would suggest only a limited role for (exogenous) EPA. Besides existing effective DM and MDD therapy (as also applied in the present study), modulation of biomarkers from these pathways by other forms of add-on treatment, such as physical exercise or cognitive therapy, would then still be valuable topics for future research.

### Conclusions

In this randomized placebo-controlled trial assessing the effect of EPA supplementation in patients with DM and comorbid MDD on a wide range of biomarkers for adverse outcome, we found limited effects. Besides increases in the supplemented EPA and α-tocopherol, AA decreased and DPA increased. Effects on the HPA-axis and lipoprotein concentrations remain inconclusive. There was no evidence for effects on biomarkers of oxidative stress, inflammation, or one-carbon metabolism. Therefore, our results emphasize the need for further investigation of the alleged beneficial effects of add-on EPA supplementation in patients with DM and MDD, preferably with clinical outcome measures such as cardiovascular complication incidence, combined with adequate assessment of the lipid peroxidation process.

## Supporting Information

Checklist S1 **CONSORT 2010 checklist of information to include when reporting a randomised trial.** *We strongly recommend reading this statement in conjunction with the CONSORT 2010 Explanation and Elaboration for important clarifications on all the items. If relevant, we also recommend reading CONSORT extensions for cluster randomised trials, non-inferiority and equivalence trials, non-pharmacological treatments, herbal interventions, and pragmatic trials. Additional extensions are forthcoming: for those and for up to date references relevant to this checklist, see www.consort-statement.org.
(DOCX)

Protocol S1 **Trial Protocol.** Addition Of Eicosapentaenoic Acid To Maintenance Anti-Depressant Therapy In Diabetes Patients With Major Depressive Disorder: A Double-Blind, Placebo-Controlled Pilot Study.
(DOC)

Table S1 **Test characteristics.**
(DOCX)

Table S2 **Baseline characteristics.** ^a^ Mann-Whitney *u* test. ^b^ Defined as having nephropathy, retinopathy, diabetic foot, macrovascular complications, or neuropathy. ^c^ According to MADRS score: 9–17 mild depression, 18–34 moderate depression, and ≥35 severe depression [28]. ^d^ At 12-week follow-up. Treatment was not specified for 1 person in the EPA arm (loss to follow-up), and for 1 person in the placebo arm.
(DOCX)

Table S3 **Linear mixed model treatment×time interaction effects for biological parameters with no significant repeated measures MANOVA in diabetes patients with MDD during 12-week randomized add-on supplementation of either ethyl-eicosapentaenoic acid or placebo (n = 24).** ^a^ Log-transformed.
(DOCX)

Translation Protocol S1 (DOC)
