# Supplementary material for: Biological Effects of Add-On Eicosapentaenoic Acid Supplementation in Diabetes Mellitus and Co-Morbid Depression: A Randomized Controlled Trial
Source: PLoS One. 2012 Nov 28;7(11):e49431. doi: 10.1371/journal.pone.0049431 (PMC3509102; doi:10.1371/journal.pone.0049431)
Supplement: Protocol S1 — Trial Protocol. Addition Of Eicosapentaenoic Acid To Maintenance Anti-Depressant Therapy In Diabetes Patients With Major Depressive Disorder: A Double-Blind, Placebo-Controlled Pilot Study. (DOC) [file pone.0049431.s002.doc]

*Versie: 30 september 2004*

## (Applicant (P.I.) : Dr. François Pouwer

## Institute: VU Medisch Centrum

## Department: Medische Psychologie

## Address: Van der Boechorststraat 7, 1081 BT Amsterdam

## Phone & Fax: + 31 20 4449679 + 31 20 4448181

## E-mail: f.pouwer@vumc.nl

## Addition Of Eicosapentaenoic Acid To Maintenance Anti-

## Depressant Therapy In Diabetes Patients With Major Depressive

## Disorder: A Double-Blind, Placebo-Controlled Pilot Study.

## Participants

| Name | Role on Project | Institute | hrs/week in project | signature |
| --- | --- | --- | --- | --- |
| Vacancy | Junior researcher | VUmc | 36 |  |
| Prof dr F. Snoek | Psychologist | VUmc | 2 |  |
| Dr F. Pouwer | Psychologist | VU mc | 2 |  |
| Prof dr A. Beekman | Psychiatrist | VUmc | 2 |  |
| Prof dr R. Heine | Internist | VUmc | Advisory |  |
| Dr P. Kostense | Statistician | VUmc | Advisory |  |
| Dr M. Diamant | Internist | VUmc | Advisory |  |
| Dr J. Assies | Internist | AMC | Advisory |  |

## Scientific Abstract (max 250 words)

Depression can be regarded as a common threat to the quality of life of diabetes patients. Evidence strongly suggests that major depression is more prevalent and more persistent in diabetes patients compared to the general population. Depression in diabetes is also associated with impaired glycaemic control, an increased risk for cardiovascular complications and increased health care consumption. Effective pharmacological interventions are available to treat major depression. Yet, in only 50-60% of the depressed subjects with diabetes, pharmacotherapy does lead to remission of depression. Both depression and diabetes were found to be associated with an impaired fatty acid metabolism. Several animal and epidemiological studies have found that consumption of ω-3 fatty acids is associated with a decreased risk for depression. Three randomised controlled trials in non-diabetic subjects demonstrated that ω-3 fatty acids as add-on therapy is effective in treating major depression, whereas one trial found that monotherapy with docosahexaenoic acid is not. So far, interventions in diabetes patients are lacking. The ω-3 fatty acids have three main advantages: they are relatively inexpensive, generally well tolerated by patients and decrease the risk of cardiovascular disease. The available evidence warrants a trial with ω-3 PUFA in particular in the diabetic population, that has a high prevalence of treatment-resistant depression. The currently proposed randomised double-blind, placebo-controlled pilot study will be the first to test this hypothesis rigorously in diabetes patients suffering from current major depression and to determine underlying physiological mechanisms.

## Summary of proposed pilot research project: objectives and plan of investigation

**Depression in diabetes:** The Global Burden of Disease Study reported that major depression was the fourth leading cause of world-wide disability in 1990 and predicted that it would be the second leading cause by the year 2020.^1^ For people with diabetes, the prevention and treatment of depression may be of even greater importance, since it was concluded that the prevalence of depression is 2-3 times higher in people with diabetes when compared to the general population (both types, approximately 10-15% instead of 5%).^2^ Recent preliminary results of our Screening for Depression in Diabetes Study [DFN 2000.00.018] confirm this: 9,9% of the outpatients of the VU University Medical center appear to suffer from Major Depressive Disorder. Depression was concluded to be more prevalent, but also more persistent and treatment resistant in diabetic adults.^2^ Furthermore, depression interferes with the quality of life of people with diabetes, and also negatively affects their selfcare behaviours and glycaemic control.^3^ Likewise, depression was found to be associated with increased development of long-term complications of diabetes (particularly cardiovascular disease and retinopathy) and increased health care consumption.^4^ For example, major depression was associated with a three-fold increased the risk for coronary heart disease at 10-year follow-up in type 1 and type 2 diabetic women attending a diabetes registry, independent of traditional risk factors. Pharmacotherapy with nortriptyline hydrochloride (tricyclic antidepressant) or fluoxetine (SSRI) was found to be an effective for treating major depression in diabetes patients compared to placebo, and successful treatment of depression was found to be associated with improvement of glycaemic control.^5^ Yet, a considerable percentage of diabetic subjects receiving the antidepressant drug did *not* achieve remission of the major depression (nortriptyline: 43% and fluoxetine 52%).^5^ This led us to the conclusion that we need to search for means to further improve the treatment of depression in patients with diabetes.

## ω-3 polyunsaturated fatty acids (PUFAS) and depression: Recent studies have reported depletions of ω-3 PUFA's among depressed patients.^6,7^ A diet higher in ω-3 PUFAs was associated with less depression.^7^ This did not simply reflect loss of appetite due to more severe depression, as the depressed patients did not differ significantly from controls in absolute total energy intake.^7^ In humans, fatty fish is the major source of ω-3 PUFAs (eicosapentaenoic acid (EPA) and docosahexaemoic acid DHA). In countries where fish consumption is high, the prevalence of depression appeared to be significantly lower;^8^ likewise, depression was associated with a lower percentage of ω-3 PUFA in plasma phospholipids.^9^ Four randomized controlled trials have recently tested the effect of ω-3 PUFA in depressed patients. The first study found no significant effect of DHA monotherapy (2 gram/day) during 6 weeks compared to placebo in 36 subjects with major depression.^10^ In second study, 20 patients with major depressive disorder participated in a 4-week double blind trial, comparing placebo with Ethyl-EPA, in addition to ongoing antidepressant therapy.^11^ Ethyl-EPA had significant beneficial effects on core depressive symptoms such as depressed mood, guilt feelings and worthlessness as well as insomnia. Depressed patients who were treated with Ethyl-EPA had a mean reduction of Hamilton depression scale score of 12.4 points, compared with 1.6 in patients receiving placebo. No clinically relevant side effects were reported.^11^ In a third, larger randomized controlled trial, 70 patients with *treatment resistant* major depression were randomized on a double blind basis to placebo or to 1 gram/day, 2 gram/day or 4 gram/day of Ethyl-EPA in addition to unchanged antidepressant medication.^12^ It was found that 69% of the patients with 1-gram/day Ethyl-EPA achieved a statistically significant and clinically relevant reduction of 50% on the Hamilton Depression Rating Scales, compared to 25% of the patients in the control group. However, the 2 gram/day group showed little evidence of efficacy, whereas the 4 gram/day group showed non-significant trends toward improvement. A fourth double-blind placebo controlled trial compared the effects of menhaden fish oil (rich in both EPA and DHA) in 28 patients with major depressive disorder, in addition to usual treatment.^13^ The ω-3 capsules contained 4.4 gram EPA/day and 2.2 gram DHA/day added with 2mg/g tocopherols as antioxidants. Results showed that after 8 weeks, subjects in the ω-3 group had significantly lower scores on the Hamilton Rating Scale for Depression compared to those in the placebo group.

**Potential mechanisms.** Fatty acids are important components of cell membranes and unsaturated fatty acids in particular determine membrane fluidity and, therefore, important membrane functions such as electrical signaling, receptor sensitivity (e.g. for insulin), and neurotransmitter release.^14^ Alterations in membrane viscosity can influence various steps in the metabolism of serotonin (5-HT), that plays a key role in the pathophysiology of depression. Lower serotonergic activity has been well established in the pathophysiology of depression. Each step in biogenic amine function, including neurotransmitter synthesis, degradation, release, reuptake, and binding is potentially influenced by the membrane composition, in particular of phospholipids and essential fatty acids.^14^ Decreased plasma membrane fluidity was associated with impaired 5-HT transport by endothelial cells, which might account for symptoms of depression.^14^ Another major function of ω-3 PUFA involves their role in the metabolism of eicosanoids and cytokines. Eicosanoids derived from the ω-3 PUFA have 10-100 fold less biological potency for inducing cellular responses than those derived from AA (ω-6 PUFA) and are therefore usually associated with decreased inflammatory responses. ω-3 PUFA consumption was found to decrease secretion of inflammatory cytokines that can provoke symptoms of depression.^14^

**Side effects of ω-3 PUFA in diabetes patients:** One advantage of ω-3 PUFA is that they are generally well tolerated by patients. Adverse effects from ω-3 supplementation are concluded to be largely related to increased intestinal gas with high doses of fish oil (10 gram/day), whereas doses of

< 3 gram/day were well tolerated and safe.^14^ Recent reviews concluded that glucose metabolism is not likely to be adversely affected by the use of ω-3 PUFA supplements.^14^ In fact, beneficial side effects can be expected as a large body of evidence consistently demonstrates that both EPA and DHA lower serum triglycerides and also reduce the risk for cardiac events (e.g. death, non-fatal myocardial infract and nonfatal stroke) and decrease the progression of atherosclerosis in coronary patients.^15^ The American Heart Association therefore recommends to people without documented coronary heart disease to eat a variety of (preferably oily) fish at least twice a week. Patients with established coronary heart disease are advised to consume 1 gram of long chain ω-3 PUFA per day.^15^

**Objectives:** To conduct a randomised trial to determine the antidepressant efficacy of EPA (1 gram/day) in diabetic patients with current major depressive disorder. A second aim is to investigate the effects of treatment and depression improvement on glycemic control. If EPA appears to be effective, a third innovative aim will be to determine for biomarkers for successful treatment with EPA (in which patients is EPA an effective antidepressant and in which patients not?).

**Methodology, setting:** This pilot randomised controlled trial will be embedded within the Institute for Cardiovascular Research VUmc (IcaR-VU), Amsterdam, the Netherlands and will be a follow up study of the research-project DFN 2000.00.018 (2002-2005): Depression screening in diabetes outpatient clinics. Point prevalence of major depression and the impact of case-finding on the course of depression and glycaemic control. In that multicenter study it is tested whether screening for depression, with feedback of depression diagnosis to the patients and their physicians, has beneficial effects on the mood of depressed subjects. For the present study, subjects with current major depression will be recruited from the group of approximately 2000 diabetes patients who then have completed the follow up measurement of the “Screening for depression in diabetes” trial.

**Inclusion criteria:** Inclusion criteria for the present study are: 1) 18-75 years old 2) having diabetes 3) current diagnosis of major depressive disorder and 4) signed informed consent. Moreover, all patients should be receiving antidepressant treatment. Exclusion criteria are: 1) psychotic features 2) history of (hypo) mania 3) an average consumption of fish higher than 2 servings per week.

**Design:** The proposed study is an 10-week, parallel group, double blind addition of eicosapentaenoic acid (EPA 1 gram/day) or placebo to ongoing anti-depressant therapy. The EPA, derived from 96% pure fish oil and stabilized with tocoferols) and matching placebo will be supplied by Minami Nutrition (Belgium). The EPA and placebo (gelatine from fish, added with tocoferols) will be provided in 500mg soft gelatin capsules. We decided to give 1 gram/day of EPA, as the results of a recent trial by Peet and Horrobin (2002) showed that the effects of EPA were dose-dependent: 1 gram/daily was found to be more effective than 2 or 4 gram/daily while a placebo was ineffective in reducing symptoms of depression. Power analyses showed a sample size of 64 subjects per group is sufficient to detect an effect size of 0.50 with a power of 0.80. The effect of the addition of EPA will be analysed using repeated measures analyses of variance with time as repeated factor, treatment (placebo or EPA) as independent factor.

**Measurements:** For all subjects, Montgomery-Asberg Depression Ratings (MADRS) will be obtained at baseline and weekly thereafter by an experienced psychiatrist or psychologist who is blind to the treatment. A MADRS score lower than 10 is regarded as a remission of depression. At baseline, all participants will be requested to complete the Dutch RIVM food frequency questionnaire, to characterise dietary intake of fat and type of fat. In this instrument, special emphasis is placed on qualification and quantification of fats, dairy products, fish, meat and dietary supplements. Moreover, variables such as age, gender, social class, body mass index, number of children, smoking habits, alcohol consumption, other drugs such as cannabis use, co-morbidity and diabetes complications will be assessed by means of a self-report questionnaire or from medical charts and if indicated used as possible confounders in the statistical analyses. Blood will be drawn by venapuncture to assess HbA_1c_ and ω-3 and ω-6 fatty acids in plasma and also ω-3 and ω-6 fatty acids erythrocyte phospholipids, as the erythrocyte membrane phospholipid composition closest reflects that of neuronal membranes. Polyunsaturated fatty acids will be quantitatively analysed in erythrocyte membranes by gas chromatography.

## Envisioned results: Go/no go criteria for further research including future strategy

If addition of EPA to maintenance antidepressant therapy in diabetes patients with major depressive disorder appears to be effective, further research is needed into the following questions: 1) Is EPA effective as an antidepressant monotherapy? 2) Can EPA supplements be used to prevent onset of depression in diabetes? 3) What are the underlying mechanisms that explain an effect of EPA on depression? To this purpose, structural and functional changes in relevant brain areas will be quantified by innovative techniques including functional MRI and in vivo cerebral phosphorus magnetic resonance spectroscopy to determine the effects of increased EPA-intake on brain membrane phospholipid metabolism in depressed diabetes patients. The effects of EPA treatment on regional glucose metabolism in the brains of diabetes patients with major depression can also be studied using Positron Emission Tomography with 18F fluorodeoxyglucose. If EPA appears to have no effect, we need to analyse and/or discuss why this is the case and report this in the scientific literature. Further research on EPA is not needed in that case (no go criterion).

## Rationale for application

For this particular grant proposal, new findings in the field of psychiatry have been combined with expertise from the area of diabetes research, and this resulted in an untested hypothesis. Testing this innovative hypothesis may be very relevant for a considerable number of diabetes patients who are suffering from depression. We expect that the present study will help to decrease the burden of diabetes, as the results may contribute to further improvements in the prevention and/or treatment of depression in diabetes. Our hypotheses can easily be tested, since this pilot randomised controlled trial can be embedded within the follow up of the Screening for Depression in Diabetes Study. The VU University Medical Centre offers excellent opportunities to conduct this research successfully, as the expertise of leading researchers in the fields of diabetes, psychosocial aspects of diabetes, depression, and polyunsaturated fatty acids is available and will be used.

Requested budget: Euro 45.000

Duration: 18 months

## Motivation

## Junior researcher (bio-medical/psychiatrist, 12 months 0.5 Fte) 30.600 Euros

2* 128 lipid profiles (erythrocytes) 6.000 Euros ^1^

## 2* 128 lipid profiles (plasma) 6.000 Euros

## 2* 128 food frequency questionnaires 1.200 Euros

*8960 EPA supplements and 8960 placebo freely*

*(2 softgels day * 70 dagen * 64 patients)* *supplied by Minami Nutrition*

## 2* 128 Depression questionnaires 1.200 Euros

^1^ Laboratory of the Amsterdam Medical Center, Discount price.

## Contribution of the proposed project in fighting diabetes mellitus

Depression can be regarded as a common psychosocial complication of diabetes that has a tremendous, negative impact on the quality of life of these patients. Depression can also hamper self-management, thereby increasing the risk for long-term diabetes-related complications. Therefore, it is important to fight depression in diabetes. Omega-3 fatty acid metabolism was found to be disturbed in a substantial number of patients with diabetes, while psychiatric studies have shown that these polyunsaturated fatty acids can protect against depression and can be used to treat depression. To our knowledge, this study would be the first to investigate the value of ω-3 PUFA in the treatment of depression in diabetes. Furthermore, this study will be the first to study biomarkers for successful treatment of depression with EPA.

**APPENDIX: I: References and II: selection of relevant publications by applicants**

**I REFERENCES:**

1. Murray CJL et al: Alternative projections of mortality and disability by cause 1990-2020: Global Burden of Disease Study. *Lancet*

349:1498- 1504, 1997.

2. Anderson RJ et al: Prevalence of depression in adults with diabetes: an epidemiological evaluation. *Diabetes Care* 24:11069-1078, 2001.

3. Lustman PJ et al: Depression and glycemic control: a meta-analytic review of the literature. *Diabetes Care* 23:934-942, 2000.

1. Egede L et al: Comorbid depression is associated with increased health care use and expenditures in individuals with diabetes. *Diabetes*

*Care* 25:464-470, 2002.

1. Lustman PJ et al: Treatment of depression in diabetes. Impact on mood and medical outcome (review) *J Psychosom Res 53:917-924*,

2002.

6. Peet M et al: Depletion of ω-3 fatty acid levels in red blood cell membranes of depressed patients. *Biol Psychiatry* 43(5):315-319, 1998.

7. Edwards R et al: ω-3 polyunsaturated fatty acid levels in the diet and in red blood cell membranes of depressed patients. *J Affect Dis*

48:149-55, 1998.

8. Hibbeln JR: Fish consumption and major depression. *Lancet* 351, 1213, 1998.

1. Tiemeier H et al: Plasma fatty acid composition and depression are associated in the elderly: the Rotterdam Study. *Am J Clin Nutr* 78:40-

46, 2003.

1. Marangell LB et el: A double-blind, placebo-controlled study of the omega-3 fatty acid docosahexaenoic acid in the treatment of major

depression. *Am J Psychiatry* 160:996-998, 2003.

1. Nemets et al: Addition of omega-3 fatty acid to maintenance medication treatment for recurrent unipolar depressive disorder. Am J Psychiatry 159:477-479, 2002.
2. Peet M, Horrobin DF. A dose-ranging study of the effects of ethyl-eicosapentaenoate in patients with ongoing depression despite

apparently adequate treatment with standard drugs. *Arch Gen Psychiatry* 59:913-9, 2002.

13 Su KP et al: Omega-3 fatty acids in major depressive disorder. A preliminary double-blind, placebo-controlled trial. *Eur*

*Neuropsychopharmacology* 13:267-271, 2003.
14. Pouwer F et al: Association of depression with type 2 diabetes mellitus: do long-chain omega-3 polyunsaturated fatty Acids play a role?

(manuscript accepted pending revisions)

15 Kris-Etherton PM, Harris WS, Appel LJ; American Heart Association. Nutrition Committee (2002): Fish consumption, fish oil, omega-3

fatty acids and cardiovascular disease*. Circulation* 106:2747-2757.

**II SELECTION OF RELEVANT PUBLICATIONS BY APPLICANTS:**

1. Popp-Snijders C, Bilo HJ, **Heine RJ**: Fish oil and glycemic control: importance of dose. *Diabetes Care* 13(1):80-1, 1990.

2. **Heine RJ.** Dietary fish oil and insulin action in humans. *Ann N Y Acad Sci* 683:110-21,1993

3. Friedberg CE, Janssen MJFM, **Heine RJ**, Grobbee DE: Fish oil and glycemic control in diabetes. A meta-analysis. *Diabetes Care* 21 (4): 494-

500, 1998.

4.  **Pouwer F** et al: Association of depression with type 2 diabetes mellitus: do long-chain omega-3 polyunsaturated fatty Acids play a role?

(manuscript submitted, under review)

5. **Pouwer F** et al: The 12-item WBQ: an evaluation of its validity and reliability in Dutch people with diabetes. *Diabetes Care* 22:2004-2010,

1999.

6. **Pouwer F**, **Snoek FJ**: Positive association between symptoms of depression and glycemic control may not be stable across sex. *Diabet Med*

2001

7. **Pouwer F**, **Snoek FJ** et al: Monitoring psychological well-being in patients with diabetes. Effects on mood, Hba_1c_ and the patients’ evaluation

of the quality of diabetes care: a randomised controlled trial. *Diabetes Care* 2001.

8. **Pouwer F**, **Snoek FJ** et al: The Well-being Questionnaire: evidence for a three-factor structure with 12 items. *Psychol Med* 30:455-462, 2000.

9. **Snoek FJ**, **Pouwer F**, et al: Diabetes-related emotional distress in Dutch and U.S. diabetic patients: cross-cultural validity of the problem areas

in diabetes scale. *Diabetes Care* 23(9):1305-1309, 2000.

10. **Pouwer F**. et al: Rates and risks for co-morbid depression in patients with Type 2 diabetes mellitus: results from a community-based study.

*Diabetologia*. 46(7):892-8, 2003.

11. **Pouwer F** and Snoek FJ: Patients' Evaluation of the Quality of Diabetes Care (PEQD): development and validation of a new instrument. *Qual*

*Saf Health Care* 11(2):131-6, 2002.

12. **Beekman AT** et al: Review of community prevalence of depression in later life. *Br J Psychiatry* 174:307-11, 1999.

13. **Beekman AT** et al: Anxiety and depression in later life: Co-occurrence and communality of risk factors. *Am J Psychiatry* 157(1):89-95, 2000.

14.**Beekman AT** et al: Depression in survivor of stroke: a community-based study of prevalence, risk factors and consequences. *Soc Psych*

*Psychiatr Epidem* 33:463-70, 1998.

15. Penninx BW, **Beekman AT** et al: Depression and cardiac mortality: results from a community-based longitudinal study. *Arch Gen Psych*

58:221-7, 2001

16. **Diamant M** et al: Elevated numbers of tissue-factor exposing microparticles correlate with components of the metabolic syndrome in

uncomplicated type 2 diabetes. *Circulation* 2002;106:2442-2447

17. **Assies J**. et al: Significantly reduced docosahexaenoic and docosapentaenoic acid concentrations in erythrocyte membranes from

schizophrenic patients compared with a carefully matched control group. *Biol Psychiatry* 15;49(6):510-22, 2001.

18. **Assies J** et al: Fatty acids and homocysteine levels in patients with recurrent depression: an explorative pilot study. *Prostaglandins Leukot*

*Essent Fatty Acids*. 70(4):349-56, 2004.

19. **Assies J** et al: Effect of dehydroepiandrosterone (DHEA) supplementation on fatty acid and hormone levels in patients with X-linked

adrenoleukodystrophy. *Adv Exp Med Biol*.544:243-4, 2003.

1. Rump P, Popp-Snijders C, **Heine RJ**, Hornstra G**.** Components of the insulin resistance syndrome in seven-year-old children: relations with birth weight and the polyunsaturated fatty acid content of umbilical cord plasma phospholipids. *Diabetologia*. 45(3):349-55, 2002.

**III CURRENT GRANTS FROM THE DUTCH DIABETES RESEARCH FOUNDATION:**

Depressed subjects at the follow up in the control group of the Screening for depression Study (DFN (2000.00.018/2002-2005) can be efficiently invited to participate in the present innovative pilot study.

**1] DFN 99.005/1999-2003** **Improving coping with diabetes: efficacy of a cognitive behavioural group training for poorly controlled type 1 diabetes patients. A randomized controlled trial**. Euro 149.638,67 1 fte PhD student (AiO) and 0.6 fte diabetes nurse specialist

**2] NWO 940-35-034, DFN 98.901/1999-2004.** **Relevance of genetic predisposition and life-style factors in the pathogenesis of type 2 diabetes mellitus and cardiovascular complications**. Euro 1.130.276,89 3 fte PhD students, and research analysts, dietician.

**3] DFN 2000.00.018/2002-2005** **Depression screening in diabetes outpatient clinics. Point prevalence of major depression and the impact of case-finding on the course of depression and glycaemic control**. Euro 173.596,46 1 fte post-doc (3 years), 0.1 fte research assistant.

**4] DFN 2003.00.020** **Monitoring health-related quality of life in adolescents with type 1 diabetes prior to periodic outpatient consultation: impact on psychosocial adaptation, satisfaction with care and glycaemic control. A randomised controlled cross-over study (**Snoek et al)

**5] DFN 2001.11.012 Functional MRI activation during cognitive function in patients with type 1 diabetes: does the presence of microvascular disease affect the response during hypoglycaemia?** (Heine et al)

**6] DFN 1999.00.007 (Heine, Bakker):** **Inhibition of adenine nucleotide translocators by long-chain fatty acyl-CoA esters and features of the insulin resistance syndrome.** Budget: € 287,129; Manpower: 1 PhD student (4 yr), 1 research technician.

**7] DFN 2000.00.025 (Diamant, Heine): The role of cell-derived microparticles in atherogenesis in DM2.**

Budget: € 245,042; Manpower: 1 PhD student (4 yr), 1 research technician.

**8] DFN 2001.00.052 (Heine, Diamant): Postprandial hyperglycaemia versus dyslipidaemia in relation to different markers of vascular dysfunction in persons with type 2 diabetes and in normoglycaemic subjects, with and without cardiovascular disease**. Budget: € 225,530; Manpower: 1 PhD student, 1 research technician.

**9] DFN 2002.00.001 (Heine, Eekhoff): Assessment of the heritability of different aspects of beta-cell function in normoglycaemic monozygotic and dizygotic twins**. Budget: € 230,000; Manpower: 1 PhD student (4 yr) and 1 research technician.

**10] DFN 2003.00.029 (Diamant): Pathophysiology of heart disease in type 2 diabetes: linking diet-induced changes in myocardial metabolism and insulin signaling to altered contractile function.** This project investigated the pathogenesis of diabetic cardiomyopathy in a rat model of high-fat diet induced insulin resistance. In particular, the role of different dietary interventions as well as the protective role of ω-3 PUFAs in diabetes-related heart disease will be addressed. Budget: € 255,670; Manpower: 1 PhD student (4 yr) and 1 research technician.

**Belangrijke aanvullende informatie:**

Ingevolge art. 7 van de Wet medisch -wetenschappelijk onderzoek met mensen (Staatsblad 1998, 161) is door de verrichter van het onderzoek, het VUmc, een verzekering afgesloten die de door het onderzoek veroorzaakte schade door dood of letsel van de deelnemende proefpersonen dekt. Deze verzekering is afgesloten bij Onderlinge Waarborgmaatschappij Centramed b.a. , Postbus 90504, 2509 LM
‘ s Gravenhage. De verzekeraar en de verzekering voldoen aan het Tijdelijk besluit verplichte verzekering bij medisch-wetenschappelijk met mensen (Staatsblad 1999, 298) gestelde eisen. Aan het onderzoek deelnemende proefpersonen zullen schriftelijk worden geïnformeerd over de verzekering.
