# Supplementary material for: Biological Effects of Add-On Eicosapentaenoic Acid Supplementation in Diabetes Mellitus and Co-Morbid Depression: A Randomized Controlled Trial
Source: PLoS One. 2012 Nov 28;7(11):e49431. doi: 10.1371/journal.pone.0049431 (PMC3509102; doi:10.1371/journal.pone.0049431)
Supplement: Table S1 — Test characteristics. (DOCX) [file pone.0049431.s003.docx]

Table S1. Test characteristics.

| **Test** | **Intra assay variabililty** | **Limit of detection** |
| --- | --- | --- |
| ROS | *7.1 %* | 40 AU/L |
| MDA | *7.6 %* | 0.01 µmol/L |
| GSSG | *3.5 %* | 25 umol/L |
| GSH | *1.1 %* | 50 umol/L |
| ƴ-tocopherol | *4.1 %* | 28 umol/L |
| α-tocopherol | *3.7 %* | 20 umol/L |
| SOD | *4.7%* | 20 U/mL |
| CRP | *3.2 %* | 0.11 mg/L |
| IL-6 | *7.5 %* | 0.1 pg/mL |
| TNF-alpha | 7.7 % | 0.5 pg/mL |
| Homocystein | 3.0 % | 0.4 mmol/L |
| Folate | 3.5 % | 0.5 ng/mL |
| Vitamin B_12_ | 6.0 % | 50 pg/mL |
| Fatty acids | 3.7 % | 0.5 mmol/l |
| LDL cholesterol | *1.3 %* | 0.26 mmol/L |
| HDL | *1.3 %* | 0.13 mmol/L |
| Total cholesterol | *1.1 %* | 0.13 mmol/L |
