# Supplementary material for: Biological Effects of Add-On Eicosapentaenoic Acid Supplementation in Diabetes Mellitus and Co-Morbid Depression: A Randomized Controlled Trial
Source: PLoS One. 2012 Nov 28;7(11):e49431. doi: 10.1371/journal.pone.0049431 (PMC3509102; doi:10.1371/journal.pone.0049431)
Supplement: Table S2 — Baseline characteristics. a Mann-Whitney u test. b Defined as having nephropathy, retinopathy, diabetic foot, macrovascular complications, or neuropathy. c According to MADRS score: 9–17 mild depression, 18–34 moderate depression, and ≥35 severe depression [28]. d At 12-week follow-up. Treatment was not specified for 1 person in the EPA arm (loss to follow-up), and for 1 person in the placebo arm. (DOCX) [file pone.0049431.s004.docx]

Table S2. Baseline characteristics.

|  | **E-EPA**  **(N=12)** | **Placebo (N=12)** | **P** |
| --- | --- | --- | --- |
| Age, means ± SD (years)^a^ | 53.1 ± 13.8 | 55.0 ± 8.6 | 0.85 |
| Body Mass Index ± SD (kg/m^2^) | 29.3 ± 5.1 | 29.8 ± 4.8 | 0.80 |
| Waist Circumference ± SD (cm)^a^ | 99 ± 15 | 100 ± 14 | 0.98 |
| Women (%) | 62 | 42 | 0.32 |
| Low Educational level (%) | 23 | 42 | 0.41 |
| Working full or part time (%) | 23 | 42 | 0.41 |
| Living with a partner (%) | 54 | 75 | 0.41 |
| Current smoker (%) | 0 | 25 | 0.10 |
| Last month fish consumption ≥ 1 serving/week (%) | 23 | 33 | 0.67 |
| Type 1 diabetes mellitus (%) | 38 | 42 | 1.00 |
| Type 2 diabetes mellitus (%) | 62 | 58 | 1.00 |
| One or more diabetes complications (%)^b^ | 38 | 17 | 0.38 |
| Treatment with diet (%) | 46 | 17 | 0.20 |
| Treatment with oral blood glucose lowering drugs (%) | 54 | 42 | 0.54 |
| Treatment with insulin (%) | 77 | 83 | 1.00 |
| Duration of diabetes, means ± SD (years) | 11.3 ± 10.7 | 18.1 ± 12.4 | 0.19 |
| HbA_1C_, means ± SD (%) | 6.9 ± 1.1 | 6.9 ± 1.1 | 0.91 |
| MADRS score, means ± SD | 26.3 ± 8.2 | 26.4 ± 8.7 | 0.97 |
| Depression severity mild (%)^c^ | 7.7 | 16.7 | 0.59 |
| Depression severity moderate (%)^c^ | 84.6 | 58.3 | 0.20 |
| Depression severity severe (%)^c^ | 7.7 | 25.0 | 0.32 |
| Using tricyclic antidepressant (%)^d^ | 17 | 0 | 0.48 |
| Using Selective Serotonin Reuptake Inhibitor^d^ | 75 | 91 | 0.64 |
| Using noradrenergic and specific serotonergic antidepressant^d^ | 8 | 9 | 1.00 |

^a^ Mann-Whitney *u* test.
^b^ Defined as having nephropathy, retinopathy, diabetic foot, macrovascular complications, or neuropathy.
^c^ According to MADRS score: 9-17 mild depression, 18-34 moderate depression, and ≥ 35 severe depression [28].
^d^ At 12-week follow-up. Treatment was not specified for 1 person in the EPA arm (loss to follow-up), and for 1 person in the placebo arm.
