# Supplementary material for: Biological Effects of Add-On Eicosapentaenoic Acid Supplementation in Diabetes Mellitus and Co-Morbid Depression: A Randomized Controlled Trial
Source: PLoS One. 2012 Nov 28;7(11):e49431. doi: 10.1371/journal.pone.0049431 (PMC3509102; doi:10.1371/journal.pone.0049431)
Supplement: Table S3 — Linear mixed model treatment×time interaction effects for biological parameters with no significant repeated measures MANOVA in diabetes patients with MDD during 12-week randomized add-on supplementation of either ethyl-eicosapentaenoic acid or placebo (n = 24). a Log-transformed. (DOCX) [file pone.0049431.s005.docx]

Table S3. Linear mixed model treatment×time interaction effects for biological parameters with no significant repeated measures MANOVA in diabetes patients with MDD during 12-week randomized add-on supplementation of either ethyl-eicosapentaenoic acid or placebo (n=24).

| **Parameter** | **Estimate** | **SE** | **95% CI** | **t** | **P** |
| --- | --- | --- | --- | --- | --- |
| CRP, mg/l^a^ | 0.10 | 0.31 | -0.54 – 0.75 | 0.33 | .745 |
| IL-6, pg/ml^a^ | -0.06 | 0.21 | -0.50 – 0.38 | -0.29 | .775 |
| TNF-α, pg/ml^a^ | -0.03 | 0.12 | -0.28 – 0.22 | -0.23 | .819 |
| AUC_g_ | -151.25 | 109.95 | -383.21 – 80.71 | -1.38 | .187 |
| AUC_i_ | -114.18 | 70.54 | -262.70 – 34.35 | -1.62 | .123 |
| Vitamine B_12_, pg/ml^a^ | 0.08 | 0.05 | -0.01 – 0.18 | 1.77 | .091 |
| Folate, nmol/l^a^ | 0.11 | 0.11 | -0.12 – 0.34 | 0.99 | .329 |
| Homocystein, µmol/l | 0.42 | 1.52 | -2.74 – 3.58 | 0.28 | .785 |

^a^ Log-transformed
