# Supplementary material for: Biological Effects of Add-On Eicosapentaenoic Acid Supplementation in Diabetes Mellitus and Co-Morbid Depression: A Randomized Controlled Trial
Source: PLoS One. 2012 Nov 28;7(11):e49431. doi: 10.1371/journal.pone.0049431 (PMC3509102; doi:10.1371/journal.pone.0049431)
Supplement: Translation Protocol S1 — (DOC) [file pone.0049431.s006.doc]

**Translation**

**Important additional information:**

Pursuant to article 7 of the Law medical scientific research with humans (Staatsblad 1998, 161), the performer of the research, the VUmc, insured any damage by death or injury to the participants. This insurance is covered by Onderlinge Waarborgmaatschappij Centramed b.a., Postbus 90504, 2509 LM, the Hague. This insurer and insurance comply with the demands postulated in the temporary decision obligatory insurance in medical scientific research with humans (Staatsblad 1999, 298). The participants will receive written information about the insurance.
